# Combined Antegrade–Retrograde Cardioplegia for Myocardial Protection During Coronary Artery Bypass Grafting in Patients with Left Main Coronary Artery Disease: The CARDIOPROTECT-LMCAD Randomised Trial

**DOI:** 10.3390/medicina62091799

**Published:** 2026-09-18

**Authors:** Jelena Lesanovic, Milan Milojevic, Djordje Nikolic, Ivan Soldatovic, Dragana Unic-Stojanovic, Igor Zivkovic, Miroslav Milicic, Petar Vukovic, Ivana Petrovic, Sinisa Jagodic, Milovan Bojic, Slobodan Micovic

**Affiliations:** 1 Department of Anesthesiology and Intensive Care, Institute for Cardiovascular Diseases Dedinje, 11000 Belgrade, Serbia; miss.sagic@gmail.com (J.L.); djole.nikolic10@gmail.com (D.N.); sinisa.jagodic@gmail.com (S.J.); 2 Faculty of Medicine, University of Belgrade, 11000 Belgrade, Serbia; soldatovic.ivan@gmail.com (I.S.); igor88zivkovic@gmail.com (I.Z.); milicic.mima@gmail.com (M.M.); petarvuk02@gmail.com (P.V.); 3 Department of Cardiac Surgery, Institute for Cardiovascular Diseases Dedinje, Milana Tepica 1, 11000 Belgrade, Serbia; mln.milojevic@gmail.com; 4 Department of Cardiovascular Research, Institute for Cardiovascular Diseases Dedinje, 11000 Belgrade, Serbia; 5 Department of Cardiac Surgery, University Hospital Zurich, University of Zurich, 8091 Zurich, Switzerland; 6 Institute of Medical Statistics and Informatics, Faculty of Medicine, University of Belgrade, 11000 Belgrade, Serbia; 7 Department of Cardiology, Institute for Cardiovascular Diseases Dedinje, 11000 Belgrade, Serbia; petrovicivana4@gmail.com (I.P.); dedinje@ikvbd.com (M.B.); 8 Faculty of Medicine, University of Banja Luka, 78000 Banja Luka, Bosnia and Herzegovina

**Keywords:** coronary artery bypass grafting, left main coronary artery disease, myocardial protection, blood cardioplegia, high-sensitivity cardiac troponin I, cardiopulmonary bypass

## Abstract

*Background and Objectives*: Myocardial protection during on-pump coronary artery bypass grafting (CABG) limits ischaemic injury, particularly in patients with left main coronary artery disease (LMCAD), in whom severe proximal stenosis may compromise antegrade cardioplegia delivery. We investigated whether combined antegrade–retrograde blood cardioplegia reduces postoperative high-sensitivity cardiac troponin I (hs-cTnI) concentrations, a marker of perioperative myocardial injury. *Materials and Methods*: We conducted a prospective, single-centre, randomised trial involving 60 adults undergoing elective isolated on-pump CABG for significant LMCAD. Patients were assigned (1:1) to antegrade-only or combined antegrade–retrograde blood cardioplegia. Cardiac biomarkers were measured after induction and at 4, 12, 24, and 48 h after ICU admission. The primary endpoint was peak hs-cTnI within 48 h; key secondary endpoints included the hs-cTnI area under the concentration–time curve, CK-MB, immediate myocardial electrical recovery and 30-day outcomes. *Results*: All patients received the assigned cardioplegia strategy, with no crossover. Peak hs-cTnI did not differ significantly between the antegrade-only and combined groups (median 529.1 [IQR 829.9] vs. 394.1 [503.4] ng/L; *p* = 0.21), nor did the hs-cTnI area under the concentration–time curve from 4 to 48 h (12,910.6 [23,127.4] vs. 8112.2 [11,686.2] ng·h/L; *p* = 0.16). Serial hs-cTnI, CK-MB, total creatine kinase, serum creatinine, immediate myocardial electrical recovery, and 30-day outcomes also did not differ significantly. No deaths, coronary sinus injuries, or other cardioplegia-related complications occurred. *Conclusions*: In patients undergoing isolated on-pump CABG for significant LMCAD, combined antegrade–retrograde blood cardioplegia did not significantly decrease peak hs-cTnI levels compared to the antegrade-only method. The combined approach was feasible with no cardioplegia-related complications, suggesting that an individualised strategy may be preferable to a routine one. Larger prospective studies are needed to determine whether higher-risk patients may derive greater benefit.

## 1. Introduction

Coronary artery bypass grafting (CABG) remains a recommended treatment modality for patients with significant left main coronary artery disease (LMCAD), with or without concomitant multivessel coronary disease, providing improved survival over optimal medical therapy and more durable protection against spontaneous myocardial infarction and repeat revascularisation than percutaneous coronary intervention (PCI) [1,2]. Despite advances in surgical techniques and perioperative organ protection, myocardial injury remains common after CABG, underscoring the need for more effective myocardial protection during aortic cross-clamping [3]. Importantly, an increase in high-sensitivity cardiac troponin (hs-cTn) after surgery is independently associated with adverse early and late outcomes [4,5,6]. Patients with LMCAD may be particularly vulnerable because severe proximal coronary stenosis can impair cardioplegia delivery to large myocardial territories, leading to heterogeneous myocardial protection during aortic cross-clamping [3,7].

Myocardial protection strategies employ various cardioplegic solutions, but none has consistently proven superior in clinical practice. Current guidelines suggest tailoring the approach to patient characteristics, coronary anatomy, and procedural complexity, while highlighting blood cardioplegia as a strategy to minimise haemodilution and bleeding-related complications [3]. However, the optimal delivery route, particularly in complex patients such as those with LMCAD, remains uncertain. Antegrade cardioplegia provides rapid electromechanical arrest but may incompletely perfuse territories distal to severe coronary stenoses or occlusions. Retrograde delivery can improve subendocardial perfusion beyond proximal obstructions, although right ventricular protection may be incomplete when used alone [3,8]. Combined antegrade–retrograde delivery may therefore provide more homogeneous myocardial perfusion when antegrade distribution is compromised. Existing evidence largely derives from small, single-centre studies using historical surgical and perfusion protocols, leaving the effectiveness of combined cardioplegia in contemporary CABG practice uncertain [7,9,10,11,12].

Given that CABG is the most commonly performed cardiac operation worldwide, even modest improvements in myocardial protection strategies could have substantial clinical relevance [13]. To address the limited contemporary evidence, we conducted a randomised trial comparing combined antegrade–retrograde and antegrade-only blood cardioplegia in patients undergoing isolated on-pump CABG for LMCAD. The primary objective was to determine whether the combined strategy reduces perioperative myocardial injury, as assessed by the peak postoperative hs-cTn concentration within 48 h after bypass surgery.

## 2. Materials and Methods

### 2.1. Study Design and Ethics

The Combined Antegrade–Retrograde versus Antegrade Blood Cardioplegia for Myocardial Protection During Coronary Artery Bypass Grafting in Patients With Left Main Coronary Artery Disease (CARDIOPROTECT-LMCAD) study was a prospective, single-centre, randomised controlled trial conducted in the Departments of Cardiac Surgery and Cardiothoracic Anaesthesiology and Intensive Care Medicine at the Dedinje Cardiovascular Institute, Belgrade, Serbia. The Institutional Ethics Committee of the Dedinje Cardiovascular Institute approved the study design on 28 April 2021 (protocol No. 1937). The study was carried out in accordance with the principles of the Declaration of Helsinki and was retrospectively registered with the ISRCTN registry (ISRCTN10547630).

### 2.2. Patient Selection and Randomisation

Consecutive adults scheduled for elective isolated on-pump coronary artery bypass grafting (CABG) for significant LMCAD, with or without additional vessel disease, were screened between April 2023 and October 2025. Significant LMCAD was defined as angiographic left main coronary artery stenosis ≥ 50%, for which the Heart Team recommended CABG. Exclusion criteria were concomitant cardiac surgery, previous cardiac surgery, emergency or off-pump CABG, dialysis-dependent kidney failure, and any clinical condition for which the operating surgeon considered randomisation between the two myocardial protection strategies clinically inappropriate.

After providing written informed consent, participants were randomised in a 1:1 ratio using a computer-generated allocation sequence implemented via the REDCap Randomisation Module to receive either antegrade-only or combined antegrade–retrograde blood cardioplegia. The operating surgeon, anaesthesiologist and perfusionist were necessarily aware of treatment allocation, whereas participants, postoperative clinical staff, laboratory personnel and investigators responsible for outcome assessment remained blinded. The study database was finalised after completion of 30-day follow-up, and analyses were subsequently performed by an independent statistician who had not participated in patient enrolment, perioperative care or outcome assessment.

### 2.3. Operative Management and Cardioplegia

Anaesthesia, CABG, cardiopulmonary bypass (CPB) and postoperative care followed standardised institutional protocols. Operations were performed through median sternotomy. After systemic heparinisation to an activated clotting time > 480 s, CPB was established with ascending aortic and single right atrial cannulation. Pump flow was maintained at ≥2.2 L/min/m^2^, mixed venous oxygen saturation at >65%, indexed oxygen delivery at >270 mL/min/m^2^ and mean arterial pressure > 50 mmHg. Goal-directed perfusion was used throughout CPB [3], with systemic temperature allowed to decrease to mild hypothermia according to institutional practice.

Blood cardioplegia was prepared by mixing oxygenated blood from the CPB circuit with a crystalloid solution in a 4:1 ratio. The final mixture contained potassium 21.5 mmol/L, magnesium 18.2 mmol/L, calcium 2.2 mmol/L, sodium 145.1 mmol/L, procaine hydrochloride 1.1 mmol/L, acetate 6.5 mmol/L, chloride 154.9 mmol/L, and bicarbonate 28.9 mmol/L, with a pH of 7.4. Cardioplegia was delivered at 4–12 °C without topical myocardial cooling. In the antegrade group, cardioplegia was administered through the aortic root at 200–300 mL/min, with root pressure maintained at ≤100 mmHg. The induction dose was 10 mL/kg, up to a maximum of 1000 mL, followed by maintenance doses of approximately 40% of the induction volume every 20–30 min. In the combined group, the same total induction dose was split between antegrade and retrograde administration in a 2:1 ratio. Retrograde cardioplegia was delivered via a coronary sinus catheter at 100–200 mL/min, with coronary sinus pressure maintained at ≤50 mmHg. Only surgeons with substantial experience in delivering retrograde cardioplegia participated in the trial. In both groups, terminal normothermic blood cardioplegia (“hot shot”; 250 mL at 37 °C) was administered antegrade at 150 mL/min for 2 min, beginning 3–5 min before aortic cross-clamp removal.

### 2.4. Study Outcomes and Follow-Up

The primary endpoint was the peak postoperative high-sensitivity cardiac troponin I (hs-cTnI) concentration during the first 48 h after CABG, defined as the highest value measured at 4, 12, 24 or 48 h after admission to the ICU.

Prespecified secondary endpoints included serial hs-cTnI concentrations and the area under the concentration–time curve from 4 to 48 h; peak and serial creatine kinase-MB concentrations; serial total creatine kinase and serum creatinine concentrations; spontaneous return of sinus rhythm; ventricular arrhythmias, defibrillation, and inotropic requirements after cross-clamp removal; mechanical ventilation duration; intensive care unit length of stay; lowest intraoperative haemoglobin level; postoperative blood loss and transfusion requirements; acute kidney injury; technical feasibility and complications of retrograde cardioplegia; and major adverse events up to 30 days.

CABG-related myocardial infarction was defined according to the Fourth Universal Definition of Myocardial Infarction as postoperative hs-cTnI > 10 times the 99th-percentile upper reference limit within 48 h, together with new pathological Q waves, angiographic evidence of new graft or native-vessel occlusion, or imaging evidence of new loss of viable myocardium or regional wall-motion abnormality [14]. Acute kidney injury was classified according to the Kidney Disease: Improving Global Outcomes criteria [15]. Suspected stroke was assessed neurologically and confirmed by computed tomography or magnetic resonance imaging. Clinical outcomes were recorded throughout hospitalisation and reassessed at 30 days via clinical evaluation or review of the electronic medical record.

### 2.5. Laboratory Measurements

Blood samples were collected at T0, after induction of anaesthesia and before initiation of cardiopulmonary bypass, and at 4, 12, 24 and 48 h after admission to the ICU. High-sensitivity cardiac troponin I (hs-cTnI) was measured in the Department of Clinical Biochemistry using the Access hsTnI assay on a UniCel DxI 600 immunoassay analyser (Beckman Coulter, Brea, CA, USA) and reported in ng/L. Peak hs-cTnI and CK-MB were defined as the highest concentrations measured at any of 4, 12, 24 or 48 h after admission to the ICU.

### 2.6. Statistical Analysis

The sample-size calculation assumed a mean peak postoperative hs-cTnI concentration of 570 ng/L and a standard deviation of 190 ng/L, and considered a 30% relative reduction clinically relevant. With a two-sided α of 0.05 and 90% power, 26 patients were required per group. Accounting for approximately 10% attrition, the planned sample size was 60 patients.

Analyses were conducted according to the intention-to-treat principle. Continuous variables were presented as mean ± standard deviation or as median (interquartile range), depending on their distribution, and categorical variables as numbers and percentages. Normally distributed continuous variables were compared using the independent-samples Student’s *t*-test, and non-normally distributed variables using the Mann–Whitney U-test. Categorical variables were compared using Pearson’s χ^2^ test or Fisher’s exact test, as appropriate.

The primary endpoint, peak postoperative hs-cTnI, was defined as the highest concentration measured at 4, 12, 24, or 48 h after ICU admission and was compared between groups using the Mann–Whitney U test. The sample-size calculation was based on the best available estimates at protocol development; because the observed hs-cTnI distribution was markedly right-skewed, a non-parametric analysis was considered more appropriate for the primary endpoint. Serial hs-cTnI, CK-MB, creatine kinase and serum creatinine concentrations were compared separately at each prespecified time point using the Mann–Whitney U test. The hs-cTnI area under the concentration–time curve from 4 to 48 h was calculated using the trapezoidal method, and the areas were compared between groups using the Mann–Whitney U test. Secondary analyses were exploratory, and no adjustment was made for multiple comparisons. All tests were two-sided, with *p* < 0.05 considered statistically significant. Analyses were performed using IBM SPSS Statistics version 29.0 (IBM Corp., Armonk, NY, USA).

## 3. Results

### 3.1. Study Population

Between April 2023 and October 2025, 96 patients were assessed for eligibility; 36 were excluded (13 did not meet the inclusion criteria, 2 declined participation, and 21 were excluded for other reasons), leaving 60 patients who were randomised to antegrade-only or combined antegrade–retrograde blood cardioplegia (30 per group) (Figure S1). All patients received the assigned intervention, with no crossover or other deviations from the randomised treatment, and all study participants completed the 30-day follow-up (Table 1).

Baseline demographic, clinical, anatomical, echocardiographic and laboratory characteristics, including the extent of coronary disease, were broadly balanced between groups, although left ventricular end-diastolic diameter was larger in the antegrade group (53.1 ± 7.3 vs. 49.6 ± 5.6 mm; *p* = 0.042) (Table 1 and Table 2).

### 3.2. Operative Characteristics

Retrograde cardioplegia was successfully delivered in all patients assigned to the combined group, without coronary sinus injury or other cardioplegia-related complications. Compared with antegrade-only cardioplegia, combined delivery used a lower antegrade volume (median 725 mL [IQR 388] vs. 1300 mL [IQR 386]; *p* < 0.001) and a median retrograde volume of 400 mL (IQR 200). Total cardioplegia volume was similar (1200 mL [IQR 500] vs. 1300 mL [IQR 336]; *p* = 0.30) (Table 3).

Aortic cross-clamp time was numerically shorter in the combined group (60.5 ± 23.2 vs. 72.4 ± 24.6 min; *p* = 0.060), whereas CPB duration did not differ significantly (88.9 ± 32.5 vs. 99.3 ± 29.7 min; *p* = 0.20). Conduit use, the lowest haemoglobin level during CPB, and the lowest CPB temperature were also similar between the groups (Table 4).

### 3.3. Troponin Levels

Peak postoperative hs-cTnI concentration did not differ significantly between the antegrade and combined groups (median 529.1 ng/L [IQR 829.9] vs. 394.1 ng/L [IQR 503.4]; *p* = 0.21). Likewise, the hs-cTnI area under the concentration–time curve from 4 to 48 h did not differ significantly (12,910.6 ng·h/L [IQR 23,127.4] vs. 8112.2 ng·h/L [IQR 11,686.2]; *p* = 0.16). Serial hs-cTnI concentrations peaked within 12 h and declined thereafter, with no significant between-group differences at any measured time point (Table 5).

### 3.4. Secondary Outcomes

Peak CK-MB levels did not differ significantly between the groups (42.0 IU/L [IQR 27.0] vs. 36.5 IU/L [IQR 15.8]; *p* = 0.37). Additionally, serial measurements of CK-MB, total creatine kinase, and serum creatinine concentrations were comparable (Table 5). Immediate myocardial electrical recovery was likewise comparable between groups. Spontaneous sinus rhythm returned in 70.0% of patients receiving antegrade cardioplegia and in 66.7% of those receiving combined cardioplegia (*p* > 0.99). Electrical defibrillation was required in 20.7% and 30.0%, respectively (*p* = 0.55), with no significant differences in ventricular fibrillation, ventricular tachycardia, atrioventricular block or temporary pacing (Table 3).

Mechanical ventilation was shorter in the combined group (6.0 h [IQR 2.8] vs. 8.5 h [IQR 5.0]; *p* = 0.010), whereas ICU stay, transfusion requirements and reoperation for bleeding did not differ significantly. No deaths occurred within 30 days. Myocardial infarction occurred in two patients in each group, and the composite major adverse event endpoint occurred in two patients in the antegrade group and three in the combined group (Table 4).

## 4. Discussion

In this randomised trial of patients undergoing isolated on-pump CABG for significant LMCAD, combined antegrade–retrograde blood cardioplegia did not significantly reduce peak hs-cTnI levels compared with antegrade-only blood cardioplegia. No statistically significant differences were observed in peak postoperative hs-cTnI, the hs-cTnI area under the concentration–time curve from 4 to 48 h, peak CK-MB, serial CK-MB concentrations, total creatine kinase or serum creatinine. Immediate electrical recovery and 30-day clinical outcomes were also similar between groups. Combined cardioplegia was technically feasible in all assigned patients, with no crossover, coronary sinus injury or other delivery-related complications.

Postoperative hs-cTnI was selected as the primary endpoint because it provides a sensitive, quantitative assessment of perioperative myocardial injury [16], and the magnitude of its postoperative elevation is independently associated with adverse outcomes after cardiac surgery [4,5,17]. In the setting of CABG, however, troponin release reflects the cumulative myocardial damage incurred throughout the procedure rather than the effects of cardioplegia alone. Potential contributors include ischaemia during aortic cross-clamping, the adequacy of cardioplegic arrest and maintenance, reperfusion injury, cardiac manipulation, systemic inflammation and graft- or native-vessel-related ischaemia [18]. Troponin elevations must therefore be interpreted within the broader clinical context, including haemodynamic, electrocardiographic or imaging evidence of myocardial ischaemia [19]. The recent EACTS expert consensus document emphasised that excessive postoperative troponin elevation is almost universal after cardiac surgery and varies with assay, sampling time, and procedural complexity [20]. Consistent with this, the VISION Cardiac Surgery study identified hs-cTnI concentrations associated with increased 30-day mortality that were substantially higher than the thresholds incorporated into the EXCEL/SCAI and ISCHEMIA definitions [21,22]. Accordingly, hs-cTnI in this trial should be interpreted as a continuous measure of the overall extent of myocardial injury. The absence of a statistically significant between-group difference suggests that combined delivery did not produce a detectable reduction in global postoperative myocardial injury under the conditions of this trial; however, it neither establishes equivalence nor excludes a smaller treatment effect.

Additionally, several aspects of the present study may explain this result. Both groups received comprehensive contemporary myocardial protection, including cold-blood cardioplegia, regular redosing, terminal warm-blood reperfusion, and goal-directed CPB management. Because myocardial protection reflects the combined effects of solution, temperature, timing, delivery route, systemic perfusion and reperfusion, these measures may have provided effective baseline protection and limited the incremental benefit of adding retrograde delivery [3,23,24,25]. Importantly, the cohort was anatomically complex: almost half of the patients had LMCAD with three-vessel disease, whereas isolated LMCAD was uncommon. Recruitment was slower than originally anticipated. Despite contemporary guideline recommendations supporting CABG as a preferred revascularisation strategy for appropriately selected patients with significant left main disease [26], many patients with less complex or isolated LMCAD were treated percutaneously at referring centres and were therefore never referred for surgical assessment. Consequently, the population reaching our institution was enriched for more extensive coronary disease, which is reflected by the high prevalence of concomitant multivessel disease in the present cohort. It remains unclear whether this complexity affects the efficacy of combined cardioplegia, as the success of antegrade protection depends not only on the extent of coronary anatomy but also on factors such as lesion location, chronic occlusions, collateral circulation, ventricular function, and ischaemic duration. The severity of left main obstruction may also be relevant, as patients with near-occlusive disease could potentially gain more from supplemental retrograde delivery, especially when antegrade cardioplegia distribution is significantly impaired. The present study was not designed to analyse this subgroup, and this hypothesis needs prospective assessment. These interconnected variables are difficult to standardise or fully assess in clinical trials, which could partly explain the varying results observed in previous studies. The shorter aortic cross-clamp duration in the combined group should also be considered when interpreting the biomarker results, as shorter ischaemic periods may reduce perioperative myocardial injury. The baseline difference in LVEDD highlights the residual imbalance that can occur despite randomisation in a relatively small trial.

Given this context, the current results do not justify adding retrograde cardioplegia to a standard antegrade blood cardioplegia protocol solely to reduce postoperative myocardial injury. They should not, however, be taken as evidence that retrograde delivery has no role in CABG. Its physiological rationale remains plausible when antegrade protection may be compromised, whereas retrograde cardioplegia alone may provide incomplete right ventricular protection [8,23,27]. Consistent with current guidelines, the myocardial protection strategy should be individualised based on coronary anatomy, ventricular function and anticipated duration of ischaemia [3].

The trial also provides relevant technical information. Retrograde cardioplegia was successfully delivered in all assigned patients without crossover, failed coronary sinus cannulation, or coronary sinus injury. However, these procedures were performed by surgeons with substantial experience and may not be reproducible early in the learning curve [28]. Previous studies comparing antegrade, retrograde and combined cardioplegia have yielded inconsistent results. Jegaden et al. reported metabolic benefits with combined delivery, whereas randomised studies by Jasinski, Kaukoranta, Radmehr and others found no consistent improvement in biochemical or functional myocardial recovery [9,10,11,12]. Onorati et al., in a study of patients with left main stem disease, reported more favourable biochemical and functional outcomes with combined intermittent blood cardioplegia [7]. Differences from the present trial may reflect variations in patient selection, coronary anatomy, cardioplegia composition and temperature, redosing, cross-clamp duration, and outcome assessment. Most earlier studies were conducted more than two decades ago, using historical perfusion protocols and conventional biomarkers. CARDIOPROTECT-LMCAD extends this evidence to contemporary practice in an anatomically complex, LMCAD-specific randomised cohort treated with blood cardioplegia, goal-directed CPB, terminal warm reperfusion, and serial hs-cTnI assessment. Under these conditions, combined delivery did not significantly reduce postoperative myocardial injury, supporting an individualised rather than a routine approach.

### Study Limitations

Several limitations should be acknowledged. This was a single-centre trial of 60 patients and was powered for a biochemical rather than clinical endpoint. The observed hs-cTnI distribution and variability differed from the assumptions used for sample-size estimation, potentially reducing the power to detect modest treatment effects. Although postoperative clinical staff and outcome assessors were blinded, intraoperative teams necessarily knew treatment allocation. Residual between-group imbalances, particularly in cross-clamp duration and LVEDD, may also have influenced treatment estimates. Secondary outcomes were exploratory and were not adjusted for multiplicity. Finally, hs-cTnI reflects overall perioperative myocardial injury rather than cardioplegia-related injury alone, and the findings may not be generalisable to higher-risk or more complex surgical populations.

## 5. Conclusions

Among patients undergoing isolated on-pump CABG for significant LMCAD, combined antegrade–retrograde blood cardioplegia did not significantly reduce postoperative peak hs-cTnI concentrations compared with antegrade-only blood cardioplegia. Combined delivery was technically feasible and was not associated with procedure-related complications in this experienced centre. These findings support an individualised rather than a routine approach to combined cardioplegia. Larger multicentre randomised trials are needed to determine whether patients at higher risk of inadequate myocardial protection, including those with severe coronary obstruction, prolonged ischaemic times or otherwise compromised antegrade delivery, may derive clinically meaningful benefit.

## Figures and Tables

**Table 1:** Baseline demographic and clinical characteristics.

Characteristic	Antegrade Cardioplegia (n = 30)	Combined A-R Cardioplegia (n = 30)	*p*-Value
**Demographic characteristics**			
Male sex	27 (90.0)	24 (80.0)	0.47
Age, years	65.4 ± 7.8	66.2 ± 6.3	0.65
Body mass index, kg/m^2^	27.5 ± 4.2	28.0 ± 3.7	0.62
Smoking status ^1^			
Never smoker	11 (36.7)	9 (32.1)	0.89
Former smoker	11 (36.7)	10 (35.7)	
Current smoker	8 (26.7)	9 (32.1)	
**Comorbidities and previous cardiovascular history**			
Arterial hypertension	29 (96.7)	30 (100.0)	>0.99
Diabetes mellitus	9 (30.0)	13 (43.3)	0.42
Dyslipidaemia	24 (80.0)	28 (93.3)	0.25
Peripheral vascular disease	1 (3.3)	6 (20.0)	0.10
Cerebrovascular disease	2 (6.7)	8 (26.7)	0.080
Chronic obstructive pulmonary disease	3 (10.0)	1 (3.3)	0.61
Previous myocardial infarction	15 (50.0)	8 (26.7)	0.11
Previous percutaneous coronary intervention	6 (20.0)	2 (6.7)	0.25
**Functional status and rhythm**			
New York Heart Association class			
Class I	8 (26.7)	11 (36.7)	0.91
Class II	12 (40.0)	7 (23.3)	
Class III	10 (33.3)	12 (40.0)	
Sinus rhythm	30 (100.0)	29 (96.7)	>0.99
Operative risk scores			
EuroSCORE II, %	1.13 (1.17)	1.21 (0.86)	0.56
STS predicted risk of mortality, %	0.61 (0.46)	0.69 (0.49)	0.47
STS predicted risk of mortality or major morbidity, %	4.59 (2.43)	4.73 (1.83)	0.68
**Chronic cardiovascular medication**			
Statin	24 (80.0)	28 (93.3)	0.25
ACEI, ARB or ARNI	28 (93.3)	26 (86.7)	0.67
Diuretic	13 (43.3)	14 (46.7)	>0.99
Beta-blocker	25 (83.3)	27 (90.0)	0.71
Calcium-channel blocker	6 (20.0)	13 (43.3)	0.095
Nitrate	16 (53.3)	14 (46.7)	0.80
ASA within 30 days	29 (96.7)	28 (93.3)	>0.99

Data are presented as mean ± standard deviation, median (interquartile range), or n (%). Abbreviations: A-R, antegrade–retrograde; ACEI, angiotensin-converting enzyme inhibitor; ARB, angiotensin receptor blocker; ARNI, angiotensin receptor–neprilysin inhibitor; ASA, acetylsalicylic acid; EuroSCORE II, European System for Cardiac Operative Risk Evaluation II; IQR, interquartile range; SD, standard deviation; STS, Society of Thoracic Surgeons. ^1^ Smoking status was available for 30 patients in the antegrade group and 28 in the combined group.

**Table 2:** Baseline coronary anatomical, echocardiographic and laboratory characteristics.

Characteristic	Antegrade Cardioplegia (n = 30)	Combined A-R Cardioplegia (n = 30)	*p*-Value
**Extent of coronary artery disease**			
LM only	1 (3.3)	3 (10.0)	0.70
LM + 1-vessel disease	7 (23.3)	5 (16.7)	
LM + 2-vessel disease	8 (26.7)	9 (30.0)	
LM + 3-vessel disease	14 (46.7)	13 (43.3)	
**Echocardiographic characteristics**			
Left ventricular ejection fraction, %	54.3 ± 6.9	54.8 ± 6.2	0.77
Left ventricular end-systolic diameter, mm	34.9 ± 6.2	32.5 ± 3.7	0.073
Left ventricular end-diastolic diameter, mm	53.1 ± 7.3	49.6 ± 5.6	0.042
**Mitral regurgitation**			
None	1 (3.3)	4 (13.3)	0.41
Trivial	17 (56.7)	16 (53.3)	
Mild	12 (40.0)	9 (30.0)	
Moderate	0 (0.0)	1 (3.3)	
**Aortic regurgitation**			
None	21 (70.0)	22 (73.3)	0.62
Trivial	6 (20.0)	8 (26.7)	
Mild	2 (6.7)	0 (0.0)	
Moderate	1 (3.3)	0 (0.0)	
**Aortic stenosis**			
None	28 (93.3)	26 (86.7)	0.39
Trivial	1 (3.3)	1 (3.3)	
Mild	1 (3.3)	3 (10.0)	
Moderate	0 (0.0)	0 (0.0)	
**Tricuspid regurgitation**			
None	5 (16.7)	5 (16.7)	0.96
Trivial	22 (73.3)	22 (73.3)	
Mild	2 (6.7)	0 (0.0)	
Moderate	1 (3.3)	3 (10.0)	
**Laboratory characteristics**			
Haemoglobin, g/dL	13.5 ± 1.7	13.9 ± 1.8	0.38
Haematocrit, %	39.7 ± 4.9	41.2 ± 3.5	0.16
Erythrocyte count, ×10^12^/L	4.56 ± 0.60	4.74 ± 0.43	0.18
Leucocyte count, ×10^9^/L	7.05 ± 1.63	7.29 ± 1.73	0.57
Platelet count, ×10^9^/L	224.5 ± 50.6	234.9 ± 57.8	0.46
Activated partial thromboplastin time, s	32.1 ± 7.4	29.9 ± 5.3 ^1^	0.20
International normalized ratio	1.08 ± 0.10	1.05 ± 0.07	0.17

Data are presented as mean ± standard deviation or n (%). Abbreviations: A-R, antegrade–retrograde; LM, left main coronary artery; SD, standard deviation. ^1^ Activated partial thromboplastin time was available for 30 patients in the antegrade group and 29 patients in the combined group.

**Table 3:** Cardioplegia administration and immediate myocardial recovery.

Variable	Antegrade Cardioplegia (n = 30)	Combined A-R Cardioplegia (n = 30)	*p*-Value
**Cardioplegia administration**			
Antegrade cardioplegia volume, mL	1300 (1113–1499)	725 (600–988)	<0.001
Retrograde cardioplegia volume, mL	0 (0–0)	400 (300–500)	<0.001
Total cardioplegia volume, mL	1300 (1163–1499)	1200 (1000–1500)	0.30
**Immediate myocardial recovery after aortic unclamping**			
Spontaneous return of sinus rhythm	21 (70.0)	20 (66.7)	>0.99
Ventricular fibrillation	6 (20.0)	9 (30.0)	0.55
Ventricular tachycardia	2 (6.7)	0 (0.0)	0.49
Complete atrioventricular block	1 (3.3)	0 (0.0)	>0.99
Temporary pacing rhythm	0 (0.0)	1 (3.3)	>0.99
Electrical defibrillation required	6 (20.7) ^1^	9 (30.0)	0.55

Data are presented as median (interquartile range) or n (%). Abbreviations: A-R, antegrade–retrograde. ^1^ Defibrillation status was documented for 29 of 30 patients in the antegrade group. The percentage was calculated using the available-case denominator.

**Table 4:** Operative characteristics and early postoperative outcomes.

Characteristic	Antegrade Cardioplegia (n = 30)	Combined A-R Cardioplegia (n = 30)	*p*-Value
**Operative characteristics**			
Unstable angina	5 (16.7)	7 (23.3)	0.75
Radial artery graft	3 (10.0)	4 (13.3)	>0.99
Right internal mammary artery	0 (0.0)	3 (10.0)	0.24
Total arterial revascularization	1 (3.3)	3 (10.0)	0.61
Aortic cross-clamp time, min	72.4 ± 24.6	60.5 ± 23.2	0.060
Cardiopulmonary bypass time, min	99.3 ± 29.7	88.9 ± 32.5	0.20
Lowest haemoglobin during CPB, g/dL	8.99 ± 1.19	9.16 ± 1.18	0.58
Lowest CPB temperature, °C	32.6 ± 0.7	32.9 ± 0.7	0.086
**In-hospital outcomes**			
Mechanical ventilation, h	8.5 (5.0)	6.0 (2.8)	0.010
Intensive care unit stay, h	25.0 (46.3)	21.5 (24.3)	0.29
Intraoperative PRBC transfusion	6 (20.0)	2 (6.7)	0.25
Any PRBC transfusion	10 (33.3)	6 (20.0)	0.38
Reoperation for bleeding	1 (3.3)	0 (0.0)	>0.99
**30-day clinical outcomes**			
All-cause mortality	0 (0.0)	0 (0.0)	—
Myocardial infarction	2 (6.7)	2 (6.7)	>0.99
Rehospitalisation	2 (6.7)	0 (0.0)	0.49
Major adverse cardiovascular events (MAE) ^1^	2 (6.7)	3 (10.0)	>0.99

Data are presented as mean ± standard deviation, median (interquartile range), or n (%). Abbreviations: A-R, antegrade–retrograde; CABG, coronary artery bypass grafting; CPB, cardiopulmonary bypass; IQR, interquartile range; MAE, major adverse events; PCI, percutaneous coronary intervention; PRBC, packed red blood cells; SD, standard deviation. ^1^ The 30-day composite of major adverse events included all-cause mortality, myocardial infarction, stroke, pulmonary embolism, renal failure, intestinal infarction, or repeat revascularisation with CABG or PCI.

**Table 5:** Serial biomarkers of myocardial injury and renal function.

Biomarker and Time Point	Antegrade Cardioplegia (n = 30)	Combined A-R Cardioplegia (n = 30)	*p*-Value
**High-sensitivity cardiac troponin I, ng/L**			
Preoperative	10.00 (18.98)	6.75 (10.00)	0.25
4 h	372.24 (281.35)	335.25 (231.49)	0.44
12 h	397.33 (315.16)	268.50 (251.48)	0.16
24 h	223.60 (405.83)	160.01 (408.32)	0.39
48 h	112.05 (299.13)	93.06 (69.99)	0.15
Peak hs-cTnI ^1^	529.11 (829.86)	394.05 (503.42)	0.21
hs-cTnI AUC (4–48 h), ng·h/L ^2^	12,910.60 (23,127.40)	8112.23 (11,686.17)	0.16
**Creatine kinase–MB, IU/L**			
Preoperative	13.50 (4.75)	12.50 (4.00)	0.17
4 h	35.50 (15.50)	30.50 (13.75)	0.82
12 h	36.00 (23.75)	28.00 (20.75)	0.37
24 h	24.00 (24.00)	23.00 (14.75)	0.54
48 h	18.00 (10.25)	16.00 (13.00)	0.90
Peak CK-MB ^1^	42.0 (27.0)	36.5 (15.75)	0.37
**Creatine kinase, U/L**			
Preoperative	81.00 (95.00)	75.50 (51.50)	0.72
4 h	453.00 (225.25)	405.50 (153.00)	0.19
12 h	574.00 (317.00)	415.50 (391.25)	0.24
24 h	525.50 (259.00)	414.00 (338.25)	0.21
48 h	372.00 (275.25)	384.00 (511.50)	0.92
**Serum creatinine, µmol/L**			
Preoperative	86.20 (29.63)	87.00 (27.65)	0.60
4 h	83.25 (34.65)	79.50 (25.35)	0.23
12 h	85.85 (28.55)	76.75 (32.58)	0.11
24 h	82.65 (30.90)	75.10 (23.98)	0.26
48 h	76.70 (27.28)	73.10 (25.68)	0.24

Data are presented as median (interquartile range). Abbreviations: A-R, antegrade–retrograde; CK-MB, creatine kinase–myocardial band; hs-cTnI, high-sensitivity cardiac troponin I; ICU, intensive care unit; IQR, interquartile range. ^1^ Peak was defined as the maximum concentration measured at 4, 12, 24 or 48 h after admission to the ICU. ^2^ The hs-cTnI area under the curve was calculated using the trapezoidal method over the 4–48-h period.

## Data Availability

The data underlying this article will be shared on reasonable request to the corresponding author, subject to institutional approval and applicable data-protection regulations.

## Supplementary Materials

The following supporting information can be downloaded at: https://www.mdpi.com/article/10.3390/medicina62091799/s1, Figure S1: CONSORT 2025 flow diagram of patient enrolment, randomisation, follow-up, and analysis in the CARDIOPROTECT-LMCAD trial. Reference [29] is cited in the supplementary materials.

## Abbreviations

The following abbreviations are used in this manuscript:

AKI	Acute kidney injury
CABG	Coronary artery bypass grafting
CK	Creatine kinase
CK-MB	Creatine kinase–MB
CPB	Cardiopulmonary bypass
hs-cTnI	High-sensitivity cardiac troponin I
ICU	Intensive care unit
IQR,	Interquartile range
LMCAD	Left main coronary artery disease
LVEF	Left ventricular ejection fraction
MAE	Major adverse events
MI	Myocardial infarction
PCI	Percutaneous coronary intervention
