# Supplementary material for: Combined Antegrade–Retrograde Cardioplegia for Myocardial Protection During Coronary Artery Bypass Grafting in Patients with Left Main Coronary Artery Disease: The CARDIOPROTECT-LMCAD Randomised Trial
Source: Medicina (Kaunas). 2026 Sep 18;62(9):1799. doi: 10.3390/medicina62091799 (PMC13609466; doi:10.3390/medicina62091799)
Supplement: Supplementary file 1 [file medicina-62-01799-s001.zip › medicina-4490088-supplementary.pdf]

Figure S1: CONSORT 2025 flow diagram of patient enrolment, randomisation, follow-up, and analysis in the CARDIOPROTECT-LMCAD trial

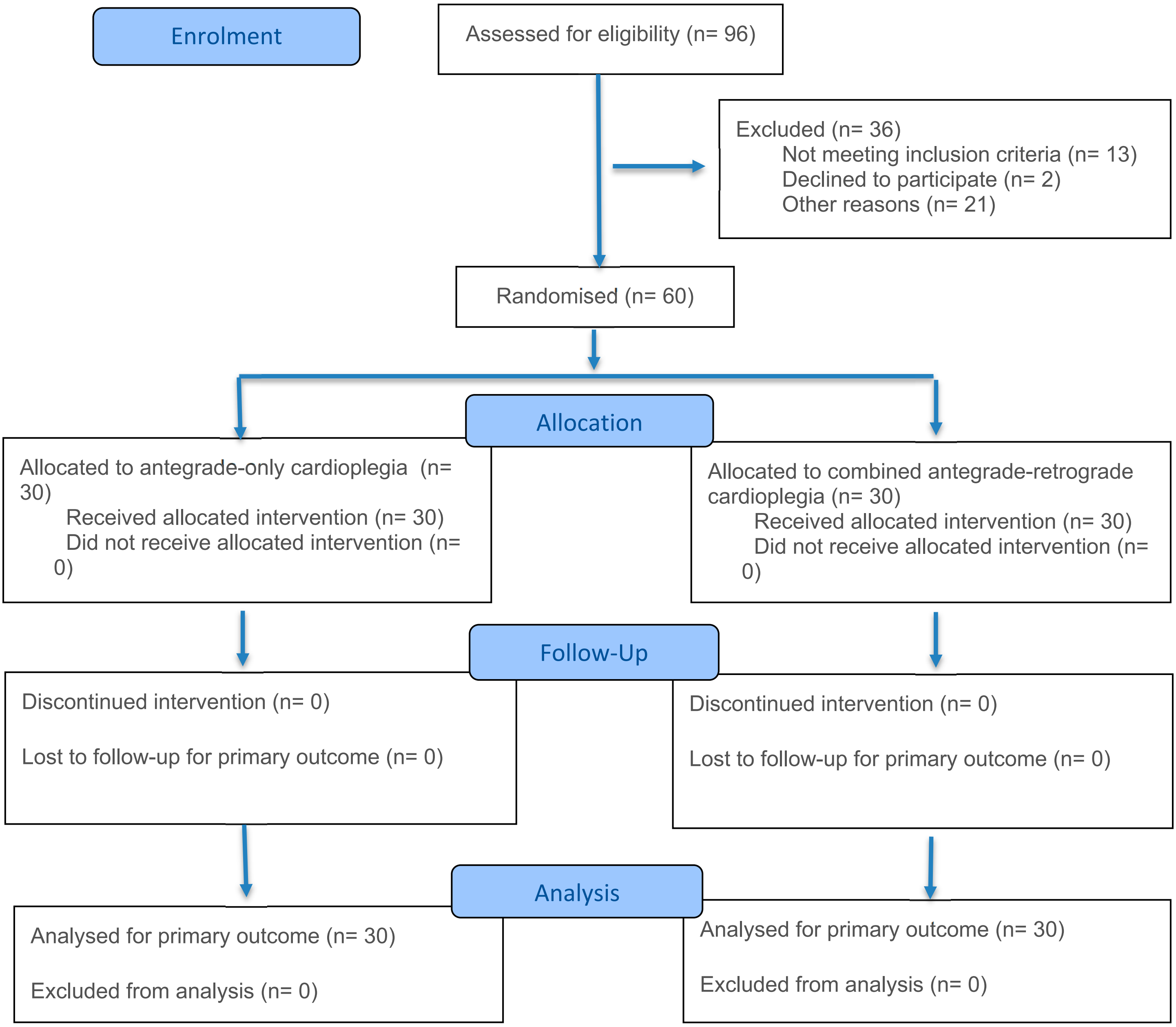

Citation: Hopewell S, Chan AW, Collins GS, Hróbjartsson A, Moher D, Schulz KF, et al. CONSORT 2025 Statement: updated guideline for reporting randomised trials. BMJ. 2025; 388:e081123. https://doi.org/10.1136/bmj-2024-081123
© 2025 Hopewell et al. This is an Open Access article distributed under the terms of the Creative Commons Attribution License (https://creativecommons.org/licenses/by/4.0/), which permits unrestricted use, distribution, and reproduction in any medium, provided the original work is properly cited.
